# Knee Muscles Isokinetic Evaluation after a Six-Month Regular Combined Swim and Dry-Land Strength Training Period in Adolescent Competitive Swimmers

**DOI:** 10.1515/hukin-2015-0121

**Published:** 2015-12-30

**Authors:** Athanasios A. Dalamitros, Vasiliki Manou, Kosmas Christoulas, Spiros Kellis

**Affiliations:** 1 Laboratory of Coaching and Sports Performance, School of Physical Education and Sport Sciences, Aristotle University of Thessaloniki, Greece; 2 Laboratory of Exercise Physiology – Ergometry, School of Physical Education and Sport Sciences, Aristotle University of Thessaloniki, Greece

**Keywords:** isokinetic testing, lower limbs, long-term evaluation, swimming

## Abstract

Previous studies demonstrated significant increases in the shoulder internal rotators’ peak torque values and unilateral muscular imbalances of the shoulder rotators after a competitive swim period. However, there are no similar data concerning the knee muscles. The purpose of the current study was to examine the effects of a six-month training period on knee flexor and extensor peak torque values, examine a possible bilateral strength deficit and evaluate the unilateral strength balance in competitive swimmers. Eleven male adolescent swimmers (age: 14.82 ± 0.45 years) were tested for concentric knee extension and flexion peak torque (60°/s) with an isokinetic dynamometer, before and after a regular combined swim and dry-land strength training period. A trend towards greater improvements in the knee extensor compared to flexor muscles peak torque was observed. Furthermore, the bilateral strength deficit remained almost unchanged, whereas unilateral strength imbalance was increased for both limbs. However, all results were non-significant (p > 0.05). According to the data presented, a six-month regular combined swim and dry-land strength training period caused non-significant alterations for all the parameters evaluated during isokinetic testing. This study highlights the fact that competitive adolescent swimmers demonstrated unilateral knee strength imbalances throughout a long period of their yearly training macrocycle.

## Introduction

Exercise testing offers valuable information regarding the determination and control of the training process. Towards this direction, isokinetic dynamometers have been used ensuring validity and reliability during muscle strength testing (Baltzopoulos and Brodie, 1989). Most of the interest in swimming has been focused on the shoulder joint. Specifically, previous studies have focused on examining muscle fatigue characteristics affecting stroke parameters (Dekerle and King, 2014), describing the strength profile of young swimmers (Batalha et al., 2012), comparing peak torque values between the two limbs of different competitive groups (Cardone et al., 1999), as well as detecting muscular strength imbalances and deficits, providing useful information regarding the possible risk of injuries (Beach and Whitney, 1992). Complementary, the effects of a swim training period were related to increased muscular strength imbalances in the shoulder rotators of young competitive swimmers (Batalha et al., 2014a), while in another study significant increments in the external / internal torque ratio during a period in which swimmers refrained from dry-land strength programs were reported (Batalha et al., 2014b).

On the other hand, legs-only swim training and dry-land strength programs are common in swimming in order to enhance performance during competition. In fact, a number of studies have indicated the supporting role of the leg kick in increasing swimming velocity, along with keeping the body in a streamlined position (Hollander et al., 1988; Deschodt, 1990; Gourgoulis et al., 2013). In addition, there are some reports on knee muscles isokinetic performance and comparison between young male and female swimmers and among competitive swimmers of different events and styles (Gerard et al., 1986; Mameletzi and Siatras, 2003; Secchi et al., 2011), or between swimmers and athletes of different sports (Siatras et al., 2004; Ozcaldiran, 2008). However, there is a lack of knowledge regarding the long-term effects of a training period on the parameters that are associated with isokinetic evaluation of knee muscles in competitive swimmers. Therefore, the purpose of this study was to examine the changes in peak torque values of the extensor and flexor muscles, a possible bilateral muscular strength deficit and evaluate the knee flexor to extensor (F/E) peak torque ratio before (pre) and after (post) a six-month regular combined swim and dry-land strength training period.

## Material and Methods

### Participants

Eleven male adolescent swimmers aged between 14 and 16 years volunteered to participate in the present study. The sample consisted of swimmers specialized in the front-crawl (three athletes), backstroke (two), butterfly (two) and breaststroke technique (two), as well as individual medley (two). In addition, eight of the participants were sprinters, while the rest were middle-distance swimmers, all participating in state and national competitions. The subjects were members of the same swimming team, tested during the same phase of their training macrocycle, and specifically at the end of the 1^st^ (0-week) and the 2^nd^ specific preparation phase (24-week), and had no previous experience in using an isokinetic dynamometer. All swimmers trained with a weekly volume between 30 and 45 km in 6 sessions, completing an average of 5 to 7 km per session, depending on the training phase and the preferred racing distance. Moreover, they were participating in the same dry-land strength training program (2 times / week), including knee extension / flexion exercises performed on resistance machines and moderate intensity plyometric training (two-foot ankle hop / countermovement jump). It should be noted that in-water strength training was not implemented during the six-month period. Swimmers with previous myoskeletal injuries or joint pathology of the lower limbs were excluded from the research. Informed consent was obtained before participation. The study was approved by the Ethical Committee of the Aristotle University of Thessaloniki and performed in accordance with the Declaration of Helsinki. Anthropometry and training characteristics of the swimmers are presented in Table 1.

### Measures and Procedures

Maximum isokinetic tests were performed on a Cybex Norm dynamometer (Lumex Corporation, Ronkonkoma, NY), previously calibrated according to the manufacturer’s instructions. Angular position and velocity were recorded at a rate of 500 Hz. At the beginning of the testing session, body composition was estimated with the bioelectrical impedance analysis (Bodystat 1500). Afterwards, participants completed a standardized 7 min warm-up on a stationary cycle-ergometer (Technogym) with pedaling cadence visually controlled at 50–60 rpm and 5 min of stretching exercises. The familiarization process for the isokinetic dynamometer included 10 sub-maximal to maximal knee extensions and flexions at the angular velocities of 180 and 60°/s (Bradic et al., 2009). Following 1 min rest, the testing protocol included 3 consecutive maximal knee extensions and flexions at the angular velocity of 60°/s. A 3 min rest period was allowed before the opposite limb was tested.

Testing was performed from the seated position, with the trunk, waist and thigh of the tested leg stabilized with restraining straps and the participants’ arms crossed over their chest. Hip flexion was adjusted electronically at 110° and the range of motion was set from 0° (full extension) to 90° (knee flexion). The participants were instructed to work as hard as possible in both directions of the movement, while standardized verbal encouragement was given at each moment. The single highest peak torque value for each trial was used for evaluation.

### Statistical Analysis

The Kolmogorov-Smirnov test was initially used to ensure data normality. The paired sample t-test was performed to compare pre and post data. Analysis was computed using the SPSSS v.21 statistical package (IBM Corp., Armonk, NY). Data are presented as mean ± *SD* and the level of statistical significance was set at *p* ≤ 0.05.

## Results

Mean *± SD* absolute peak torque values, the bilateral muscular strength deficit, as well as the knee peak torque F/E ratio for pre and post conditions are presented in Table 2. Peak torque values increased between pre and post tests for both extensor and flexor muscles. However, these changes were non-significant (5.21 to 8.67%, *p =* 0.369 to 0.650). In contrast, the peak torque F/E ratio was reduced, but also not significantly (3.35 and 2.92%, *p* = 0.597 and 0.582, for the right and left limb, respectively). Similar results were observed for the bilateral muscular strength deficit that remained almost unchanged (2.58 vs. 1.12%, *p* = 0.730 for the extensor muscles, and 2.89 vs. 1.33% for the flexor muscles, *p* = 0.767, for pre vs. post conditions, respectively).

## Discussion

To our knowledge this is the first study with a repeated-measures design that involved knee muscles isokinetic evaluation in competitive swimmers. Our findings indicated that the tested swimmers exhibited non-significant peak torque improvements in the knee flexor and extensor muscles and only a small variation in the bilateral muscular strength deficit, after a six-month regular combined swim and dry-land strength training period. In contrast, unilateral muscular strength imbalances (described with the knee F/E peak torque ratio) were recorded after the training period due to higher percentage increases of the extensor compared to flexor muscles (Table 2). These differences in strength improvements might be associated to swimming biomechanics (Secchi et al., 2011) and/or the type of a dry-land strength training program. Nevertheless, all of the aforementioned changes were non-significant (p > 0.05).

The lack of significant improvements in peak torque values can be attributed to the specific period of the annual training cycle that testing was conducted (end of the 1st – end of the 2nd specific preparation phase). It is likely that testing during the basic preparation phase, in which one of the principal purposes is to increase overall muscular strength (Maglischo, 2003), would conclude in different results. However, in this study the main focus was to evaluate lower limb isokinetic variables of a typical competitive swimming team participating in a regular combined swim and dry-land strength training program during the longest possible period of the annual training cycle.

Although there are no reports describing the optimum F/E peak torque ratio or correlating the low F/E peak torque ratio values with the occurrence of injuries in swimmers, values approaching 60% (at the angular velocity of 60°) are generally accepted for injury prevention during dynamic movements (Coombs and Garbutt, 2002), Thus, F/E peak torque ratio values presented here (51.61 to 53.44%) can be described as relatively low. Similar results are presented in the study of Secchi et al. (2011), with values ranging from 46.5 to 53.5%. Peak torque values of the latter study were also close compared to the present one, especially for the flexor muscles (204.2 to 223.9 vs. 188.52 to 209.82 Nm and 96.0 to 118.8 vs. 100.68 to 108.48 Nm, for the extensor and flexor muscles, respectively), despite the differences in the competitive level and the chronological age of the participants. In addition, a small number of female swimmers (*n* = 3) were also included in the aforementioned study. The limited available data concerning peak torque values of the lower limbs in swimmers could be attributed to the lack of specificity with the swimming movement observed during isokinetic testing (Cardone et al., 1999).

The bilateral muscular strength imbalance is generally proposed as a factor that may influence athletic performance and increase the risk of injury (Knapik et al., 1991; Yoshioka et al., 2010). Still, there is a controversy regarding the recommended percentage of the bilateral strength balance (Tol et al., 2014), with a difference of more than 10% to be characterized as abnormal (Fousekis et al., 2010). Nevertheless, these results refer to soccer players. In that respect, the bilateral strength deficit reported in the present study can be accepted as normal and, furthermore, remained almost constant (2.58 and 1.12%, for pre and post conditions, respectively). Since swimmers do not rely on the leg preference for increased propulsion, future work examining potential bilateral muscular strength imbalances of the lower limbs should focus on underlying factors such as overuse and technique (Sanders et al., 2011), especially in athletes mainly performing the breaststroke technique.

In swimming, muscle imbalances and deficits of the lower limbs have not drawn the same attention of researchers compared to the upper limbs, since the propulsion is mostly attributed to the upper body, especially during the execution of freestyle, butterfly and backstroke techniques (Batalha et al., 2014b). Moreover, the most common musculoskeletal problem in competitive swimming is the “swimmer’s shoulder” (Ciullo and Stevens, 1989; Bak, 2010). However, several aspects should be considered, highlighting the importance of aiming at the recommended bilateral and unilateral knee muscle balance via establishing dry-land strengthening programs focused on technical proficiency and with frequent testing: i) the high incidence of knee injuries in swimmers specialized in the breaststroke technique (Rovere and Nichols, 1985), ii) the fact that swimmers implement in-water training with dry-land based programs including strength machines and plyometric exercises, and iii) the explosive force production required during pushing off the wall after the turn.

An obvious limitation of the current study can be addressed regarding to the testing position. Despite the fact that the prone testing position should be more sports-specific, peak torque values attained during the sitting position are considered to be higher (Manou et al., 2003), reflecting a more accurate F/E peak torque ratio. Another limitation is related to the heterogeneous sample employed, regarding the preferred competitive swimming stroke and race distance as well as the absence of a control group. However, the main objective of this study was to examine the changes of the isokinetic parameters tested after a six-month regular combined swim and dry-land strength training period of a typical swimming team and not to determine the effectiveness of the specific training program.

## Conclusion

The male adolescent competitive swimmers tested in this study demonstrated non-significant peak torque improvements in knee flexor and extensor muscles, minimal variations in the bilateral muscular strength deficit and a lower knee F/E peak torque ratio after a six-month regular combined swim and dry-land strength training period. Since a unilateral strength imbalance was observed throughout the training period, it is suggested that specific exercises strengthening the flexor muscle group should be implemented in the training program and that testing on regular basis is needed in swimming in order to ensure the recommended F/E ratio.

## Figures and Tables

**Table 1:** Participants’ anthropometry and training characteristics

Variable	
Age (years)	14.82 ± 0.45

Anthropometric characteristics	

Body height (m)	1.78 ± 0.15
Arm span (m)	1.83 ± 0.17
Body mass (kg)	67.42 ± 6.32
Body fat percentage (%)	6.03 ± 2.50

Training characteristics	

Training experience (years)	6.63 ± 0.30
Swimming training frequency (sessions/week)	6.27 ± 0.41
Swimming training time (min/session)	102.35 ± 21.41
Dry-land strength training time (min/session)	55.16 ± 10.23

**Table 2:** Results of the isokinetic evaluation for both pre and post conditions

Variable	Pre	Post	Difference (%)	p
Knee extension				

PT 60°/s (Nm) (R)	196.57 ± 61.61	209.82 ± 45.80	6.31	0.574
PT 60°/s (Nm) (L)	188.52 ± 46.95	206.41 ± 44.22	8.67	0.369

Deficit (%)	2.58 ± 8.78	1.12 ± 10.65		0.730

Knee flexion				

PT 60°/s (Nm) (R)	102.83 ± 28.71	108.48 ± 28.95	5.21	0.650
PT 60°/s (Nm) (L)	100.68 ± 28.55	107.52 ± 29.65	6.36	0.502

Deficit (%)	2.89 ± 13.81	1.33 ± 10.32		

F/E ratio (%) (R)	53.44 ± 8.59	51.65 ± 6.85	3.35	0.597
F/E ratio (%) (L)	53.16 ± 6.51	51.61 ± 6.53	2.92	0.582

PT = peak torque; F/E ratio = flexor/extensor peak torque ratio; (R) = right limb; (L) = left limb.
